# Integrating malaria surveillance with climate data for outbreak detection and forecasting: the EPIDEMIA system

**DOI:** 10.1186/s12936-017-1735-x

**Published:** 2017-02-23

**Authors:** Christopher L. Merkord, Yi Liu, Abere Mihretie, Teklehaymanot Gebrehiwot, Worku Awoke, Estifanos Bayabil, Geoffrey M. Henebry, Gebeyaw T. Kassa, Mastewal Lake, Michael C. Wimberly

**Affiliations:** 1 Geospatial Sciences Center of Excellence, South Dakota State University, Brookings, SD USA; 2 Department of Electrical Engineering and Computer Science, South Dakota State University, Brookings, SD USA; 3 Health, Development, and Anti-Malaria Association, Addis Ababa, Ethiopia; 4 Amhara National Regional State Health Bureau, Bahir Dar, Ethiopia; 5 School of Public Health, Bahir Dar University, Bahir Dar, Ethiopia; 6 Gamby College of Medical Science, Bahir Dar, Ethiopia

**Keywords:** Malaria informatics system, Surveillance, Remote sensing, Environmental data, Risk map, Early detection, Early warning, Forecasting

## Abstract

**Background:**

Early indication of an emerging malaria epidemic can provide an opportunity for proactive interventions. Challenges to the identification of nascent malaria epidemics include obtaining recent epidemiological surveillance data, spatially and temporally harmonizing this information with timely data on environmental precursors, applying models for early detection and early warning, and communicating results to public health officials. Automated web-based informatics systems can provide a solution to these problems, but their implementation in real-world settings has been limited.

**Methods:**

The Epidemic Prognosis Incorporating Disease and Environmental Monitoring for Integrated Assessment (EPIDEMIA) computer system was designed and implemented to integrate disease surveillance with environmental monitoring in support of operational malaria forecasting in the Amhara region of Ethiopia. A co-design workshop was held with computer scientists, epidemiological modelers, and public health partners to develop an initial list of system requirements. Subsequent updates to the system were based on feedback obtained from system evaluation workshops and assessments conducted by a steering committee of users in the public health sector.

**Results:**

The system integrated epidemiological data uploaded weekly by the Amhara Regional Health Bureau with remotely-sensed environmental data freely available from online archives. Environmental data were acquired and processed automatically by the EASTWeb software program. Additional software was developed to implement a public health interface for data upload and download, harmonize the epidemiological and environmental data into a unified database, automatically update time series forecasting models, and generate formatted reports. Reporting features included district-level control charts and maps summarizing epidemiological indicators of emerging malaria outbreaks, environmental risk factors, and forecasts of future malaria risk.

**Conclusions:**

Successful implementation and use of EPIDEMIA is an important step forward in the use of epidemiological and environmental informatics systems for malaria surveillance. Developing software to automate the workflow steps while remaining robust to continual changes in the input data streams was a key technical challenge. Continual stakeholder involvement throughout design, implementation, and operation has created a strong enabling environment that will facilitate the ongoing development, application, and testing of the system.

## Background

Despite the significant progress that has been made toward reducing the global burden of malaria, this disease remains one of the most significant public health threats in sub-Saharan Africa and many other parts of the developing world [1, 2]. Access to timely and accurate information about malaria transmission is critical for control and elimination efforts. This need is particularly acute in highland and semi-arid regions where marginal environments support unstable malaria transmission and epidemics are often associated with inter-annual fluctuations in rainfall and temperature. Malaria epidemics can cause high levels of severe morbidity and mortality because populations in epidemic-prone areas typically lack acquired immunity [3]. Early indication of an emerging epidemic can provide opportunities for proactive interventions, including rapid mobilization to increase the availability of malaria drugs and other health care services in the affected regions, and timely implementation of vector-control measures such as indoor residual spraying, distribution of long-lasting insecticide-treated bed nets (LLINs), and source control of vector populations [3–5]. Conversely, knowing that an epidemic is not occurring can limit the investment of scarce resources on drugs and vector control when they are not required. In malaria elimination campaigns, the potential for resurgent epidemics is a major concern, and information about the location and timing of residual transmission hotspots is needed to facilitate rapid and effective public health responses. Given these issues, there is a strong need for innovative malaria information systems that facilitate data sharing among stakeholders and enable the use of this information to direct public health response [6–8]. To address this need, the Epidemic Prognosis Incorporating Disease and Environmental Monitoring for Integrated Assessment (EPIDEMIA) computer system was developed to support the detection and forecasting of malaria epidemics in the Amhara region of Ethiopia.

Malaria surveillance systems track the temporal trends and spatial patterns of malaria cases and deaths, providing a basis for the early detection of malaria epidemics. The standard technique for early detection involves computing a threshold value for a malaria indicator within a given geographic region based on the expected distribution under “normal” conditions as inferred from historical data [9]. Observations exceeding this threshold are interpreted as signs of a rising epidemic curve, and various rules for outbreak alerts can be defined based on the numbers and magnitudes of these exceedances. When specific information about malaria case locations is available, methods that account for both spatial and temporal clustering of cases can highlight the locations of emerging malaria hotspots [10]. By definition, early detection does not provide information about an epidemic until it is already underway. Therefore, malaria information systems are essential to facilitate rapid acquisition, processing, and sharing of data so there is adequate time to detect an incipient epidemic and implement a public health response. The use of mobile health (mHealth) technologies, such as short message service (SMS) based data transmission has been explored in various settings as a solution for increasing the speed and accuracy of malaria surveillance [11, 12]. More generally, the development of web-based data management platforms has been proposed as a critical strategy for strengthening surveillance by automating major data processing steps, enabling data access, implementing outbreak alerts, and integrating surveillance data with other relevant sources of information [6, 7].

In addition to malaria case surveillance, environmental data can also be used to predict malaria epidemics in settings where vector populations are sensitive to meteorological conditions and habitat availability. Epidemics are often associated with temperature increases in highland settings where cool temperatures typically limit the development rates of parasites and mosquitoes. In contrast, outbreaks are more affected by rainfall in warmer and more arid regions where the lack of temporary water bodies for breeding constrains the size of mosquito populations [13, 14]. Because malaria cases exhibit a lagged response of weeks or months to these climatic factors, meteorological information can provide early warning about malaria risk prior to the actual start of the epidemic [13, 15]. One significant barrier to the use of environmental data for malaria surveillance is the scarcity of in situ monitoring networks, such as meteorological stations, in many parts of the developing world [16]. Earth observations from space-borne sensors can provide relevant data on rainfall, temperature, and other climatic variables [17, 18] along with geographic information about water bodies, irrigated agriculture, wetlands, and other land cover and land use characteristics that can affect mosquito habitats and the exposure of human populations [19, 20]. Satellite-based remote sensing provides consistent and recurring measurements from nearly everywhere on the earth’s surface, and these data are used widely in research on malaria and other mosquito-borne diseases [21]. Thus, the integration of remotely-sensed environmental data with malaria surveillance provides an opportunity to expand the scope and enhance the effectiveness of malaria information systems.

There are several significant challenges that must be met to achieve the goal of integrating malaria surveillance with environmental monitoring data in a malaria information system. Whereas human case surveillance is usually conducted using a tabular database, remotely-sensed environmental data are typically obtained as gridded geospatial datasets, which must go through multiple levels of processing to *harmonize* the geospatial information. Two key steps in this process include spatial harmonization, in which the environmental grids are linked to the geographic regions for which surveillance data are tracked, and temporal harmonization, in which the various time steps used to collect data are reconciled. These procedures are complicated by the fact that various environmental datasets have different file formats, grid cell resolutions, and update frequencies. Efficient harmonization of these disparate data can be facilitated by (1) the development of a comprehensive workflow to achieve the major data processing steps, (2) the implementation of this workflow as a computer software system to facilitate automated data acquisition and processing, and (3) the creation of a web-based portal to facilitate stakeholder access to the data and to the results of outbreak detection algorithms, malaria forecasts, and other derived products.

The main objective of our project was to develop a malaria information system to support malaria early detection and forecasting in the Amhara region of Ethiopia. This paper documents the design and implementation of EPIDEMIA, a prototype system for integration of malaria surveillance with environmental monitoring data to generate operational forecasts of malaria outbreak risk. The problem is first outlined by documenting the specific datasets used in the system and describing the major workflow steps necessary to generate a unified database suitable for modelling and prediction. Although the emphasis of this paper is on system design and information processing rather than the predictive models, a general discussion of the modelling and reporting steps is provided in the context of the broader data processing workflow for completeness. Next, technical information about the workflow implementation is presented, including details about the hardware and software. Examples of the data generated by the harmonization process, along with the forecasts and reports generated by EPIDEMIA, are provided. Finally, the lessons learned in the development of this prototype are summarized and areas for future expansion and improvement of malaria information systems in general are discussed.

## Methods

### System design

A co-design workshop for the primary stakeholder groups was conducted in July 2014. The participants included public health partners from the Amhara National Regional State Health Bureau (ARHB), the Health Development and Anti- Malaria Association (HDAMA), the GAMBY College of Medical Sciences, and Bahir Dar University, as well researchers and software engineers from South Dakota State University. This workshop included a formal requirements analysis focusing on the design of the user interface, the epidemiological data upload process, automation of report generation, accessibility of the unified dataset, and system security. The following broad requirements for the EPIDEMIA system (hereafter, the system) were identified:

- User access must be provided through a password-protected web-based interface;
- The system must provide a simple and efficient interface for uploading epidemiological data, and then screening these data for errors and inconsistencies;
- The system must automatically acquire and process the most recent remotely-sensed environmental data and harmonize them with the epidemiological data to generate an integrated dataset suitable for analysis and modelling;
- Users must have access to the integrated dataset via a simple, menu-driven interface;
- The system must automatically generate early detection alerts and epidemic forecasts when new epidemiological data are uploaded; and
- The system must automatically summarize this information into standardized reports for the users.

**Fig. 1:**
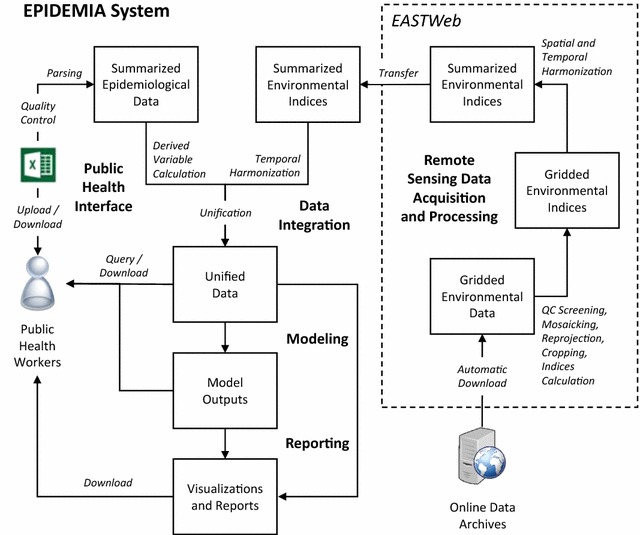
EPIDEMIA system design. This conceptual diagram describes the flow of information into, within, and out of the system. *Bold text* represents major subsystems, described in the paper, and *boxes* and *arrows* represent the main data flows

Based on these requirements, a conceptual design was created to describe the flow of information into, within, and out of the system (Fig. 1). The overarching goal was to create an informatics framework that would allow all project partners, including research scientists and public health professionals in the USA and Ethiopia, to have shared access to harmonized epidemiological and environmental data. A concurrent goal was to automate data acquisition and processing steps as much as possible, so that data could be updated in near-real time to support the implementation of early detection and early warning systems for malaria.

### Study area

**Fig. 2:**
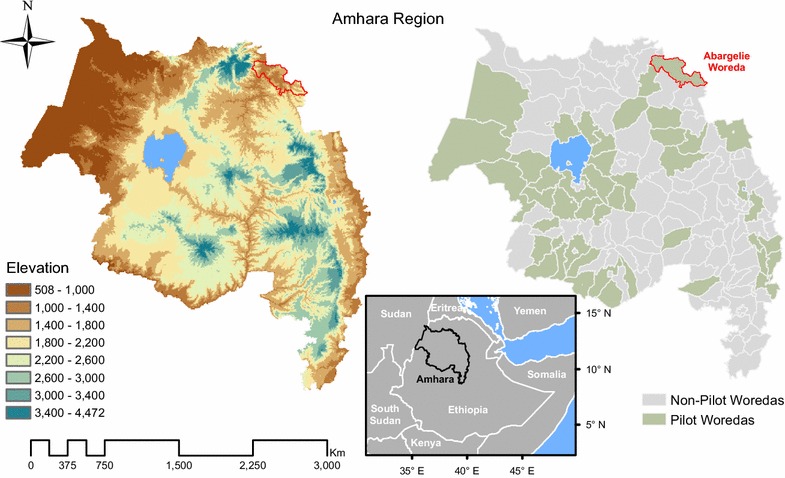
Maps of the Amhara region in Ethiopia showing elevation and woreda boundaries. The 47 pilot woredas included in this study are highlighted in *green*

The Amhara region is located in northwestern and north central Ethiopia between 9°00 and 13°45 N and 36°00 and 40°30 E (Fig. 2). Much of the terrain is mountainous, with elevations ranging from 506 to 4517 m above sea level. Mean annual rainfall varies from 770 to 2000 mm, is highest in the southwestern part of the region, and generally decreases to the east. Rainfall is highly seasonal, with the heaviest rains occurring from June through September, and dry conditions prevailing from October through February. Average annual air temperature ranges from 16 °C in the summer to 27 °C in the dry season and generally decreases with increasing elevation.

The main malaria transmission season occurs from September-December following the end of the rainy season, with a smaller peak often occuring in May–June [14, 22]. Outbreaks of malaria are often associated with climatic fluctuations and can cause high morbidity and mortality because the human population lacks immunity to the pathogen [23, 24]. The most recent regional malaria epidemic was in 2003–2005 [24, 25], and since that time malaria outbreaks have generally been smaller and more localized [22]. *Anopheles arabiensis* is the principal malaria vector [26], and *Plasmodium falciparum* and *Plasmodium vivax* are both important malaria parasites throughout the region [27]. Ethiopia, including the Amhara region, has a national malaria control programme which includes the distribution of free LLINs, targeted indoor residual spraying (IRS), rapid diagnostic tests (RDTs), and treatment with artemisinin-based combination therapy [27]. Because of unstable transmission and declining malaria incidence, low-transmission areas within the region have been targeted for malaria elimination, and elimination strategies are being evaluated through an ongoing demonstration project [28].

The region has a population of >20 M, most of whom live in rural areas and practice subsistence agriculture. Administratively, Amhara is divided into 10 *zones* and 3 *administered towns*. Each zone is divided into 7 to 27 *woredas*, or districts, which range in size from 3.7 to 7700 sq km and in estimated 2016 population from ~20 to ~384 K. Each woreda is further divided into villages called *kebeles*. There are 167 woredas and 3463 kebeles in the region, and the EPIDEMIA project currently encompasses 47 pilot woredas that were selected to encompass the most malaria-prone parts of the region (Fig. 2). Health care facilities are organized hierarchically into *primary health care units* (PHCUs). Each PHCU is comprised of five *health posts*, a *health center*, and, in some areas, a *hospital*. Health posts, the satellite facilities in the PHCU, are located in kebeles and each serves approximately 5000 people. Each health center serves approximately 25,000 people; whereas, hospitals can serve anywhere from 100,000 to 5 million people. Currently, there are 54 hospitals, 836 health centres, and 3354 health posts in Amhara.

### Datasets

#### Epidemiological data

Surveillance data is collected by the ARHB on all patients who seek treatment at health posts and health centers. These data are summarized by World Health Organization (WHO) epidemiological week and reported to the woreda health office. The data are then aggregated for the entire woreda and sent to the zonal health offices, where the data for all woredas within a zone are compiled and sent to the regional office of the ARHB. The resulting weekly data table contains one record for each *woreda*, with multiple data fields that hold information about the numbers and characteristics of malaria cases reported for that week.

Weekly counts of total patient visits are provided along with counts of total malaria cases (including clinically diagnosed as well as confirmed malaria) broken down by age and pregnancy status. Numbers of tests performed by rapid diagnostic tests (RDT) and blood film screening are also reported, along with the numbers of cases confirmed using each method. Confirmed cases are grouped into two categories: *P. falciparum* plus mixed *P. falciparum*/*P. vivax* infections (hereafter, *P. falciparum* malaria), or *P. vivax*-only infections (hereafter, *P. vivax* malaria).

Estimates of the total population living within the malarious portion of each woreda were provided by the ARHB. These estimates were derived from kebele-level population data obtained in the 2007 national census. These population numbers were updated for subsequent years by applying an estimated regional growth rate of 1.8%. Despite the assumptions that were required, these values represent the best small-area population estimates currently available for Amhara and are currently used by the ARHB for their malaria assessments. The morbidity and population data were used to calculate malaria indicator variables including malaria incidence, proportion of patients diagnosed with malaria, proportion of total cases confirmed by diagnostic tests, and proportion of positive diagnostic tests (Table 1).

**Table 1:** Malaria indicator variables calculated by the EPIDEMIA system

Variable name	Equation
Incidence of malaria	(*C* + *U*)/*P*
Incidence of confirmed malaria	*C*/*P*
Incidence of *P. falciparum* malaria	*C*_*f*_/*P*
Incidence of *P. vivax* malaria	*C*_*v*_/*P*
Proportion of patients with malaria	(*C* + *U*)/*T*
Proportion of patients with confirmed malaria	*C*/*T*
Proportion of patients with *P. falciparum* malaria	*C*_*f*_/*T*
Proportion of patients with *P. vivax* malaria	*C*_*v*_/*T*
Confirmed malaria positivity rate	*C*/*D*
*P. falciparum* malaria positivity rate	*C*_*f*_/*D*
*P. vivax* malaria positivity rate	*C*_*v*_/*D*

Summarized for each *woreda* in each epidemiological week

*C* confirmed malaria cases, *U* unconfirmed malaria cases, *P* total population living in malarious areas, *C*_*f*_ confirmed *P. falciparum* and mixed *P. falciparum*/*P. vivax* cases, *C*_*v*_ confirmed *P. vivax* cases, *T* total number of cases for all causes, including malaria, *D* total number of diagnostic tests performed

#### Environmental data

Environmental variables were obtained from remotely-sensed earth observation data products produced by the US National Aeronautics and Space Administration (NASA, Table 2). To support our goals of conducting malaria early detection and early warning, we selected products that had global coverage, free access, high data quality, and low latency (the delay between the times when raw data are collected and when processed data products are made available for use). Data on rainfall, temperature, and various spectral indices of vegetation greenness and surface moisture were used, based on previous studies that demonstrated their potential for predicting malaria outbreaks in the Amhara region [14, 22].

**Table 2:** Environmental indices calculated by the EASTWeb software

Name	Description	Source
Precipitation	Total amount of water falling from the atmosphere to the land surface (mm/week)	$\sum\limits_{d = 1}^{7} {P_{d} }$
Land surface temperature	Radiative skin temperature of the earth’s surface (°C)	$\frac{{\left( {T_{d} + T_{n} } \right)}}{2}$
Normalized difference vegetation index	Indicator of the amount of green vegetation. Can serve as a proxy for available soil moisture in water-limited environments	$\frac{{B_{2} - B_{1} }}{{B_{2} + B_{1} }}$
Soil-adjusted vegetation index	Similar to NDVI, but minimizes soil brightness influences	$\frac{{1.5 \times \left( {B_{2} - B_{1} } \right)}}{{B_{2} + B_{1} + 0.5}}$
Enhanced vegetation index	Similar to NDVI, but corrects for atmospheric distortion and ground cover below canopy	$\frac{{2.5 \times \left( {B_{2} - B_{1} } \right)}}{{B_{2} + 6 \times B_{1} - 7.5 \times B_{3} + 1}}$
Normalized difference water index	Indicator of water content in vegetation and at the soil surface	$\frac{{B_{2} - B_{5} }}{{B_{2} + B_{5} }}$
		$$\frac{{B_{2} - B_{6} }}{{B_{2} + B_{6} }}$$

Indices were calculated from various remote sensing data products as described in the text

*P*_*d*_ daily precipitation, *T*_*d*_ daytime land surface temperature, *T*_*n*_ nighttime land surface temperature, *B*_1_ MODIS band 1 (red, 620–270 nm), *B*_2_ MODIS band 2 (near infrared, 841–876 nm), *B*_3_ MODIS band 3 (blue, 459–479 nm), *B*_5_ MODIS band 5 (middle-infrared, 1230–1250 nm), *B*_6_ MODIS band 6 (middle infrared, 1628–1652 nm)

The NASA tropical rainfall measuring mission (TRMM) multi-satellite precipitation analysis (TMPA) provides gridded precipitation data for tropical and subtropical areas beginning in 1998 using data from multiple satellites and meteorological stations [29–31]. A near real-time dataset is released with a latency of less than a day and incorporates only the satellite data, while a higher quality estimate incorporating the meteorological station data is released with a latency of several months. Both real-time and research-quality products have a spatial resolution of 0.25 degrees and consist of 3-h estimates of rainfall rate. The NASA Goddard Earth Sciences Data and Information Services Center (GES DISC) summarizes these data as daily rainfall totals and releases them as separate products [32, 33].

Two datasets derived from NASA’s moderate resolution imaging spectroradiometer (MODIS) on board the Terra and Aqua satellites were also used. The LST and Emissivity 8-day 1 km dataset (MOD11A2) [34] provided daytime and nighttime land surface temperature (LST), and these values were also summarized to compute mean daily LST. The Nadir BRDF-adjusted reflectance (NBAR) 16-day 1 km dataset provided modeled reflectance values for the MODIS visible to shortwave infrared bands (MCD43B4) [35]. These products were used to calculate a variety of spectral indices related to the greenness and wetness of the Earth’s surface, including the normalized difference vegetation index (NDVI) [36], enhanced vegetation index (EVI) [37], soil-adjusted vegetation index (SAVI) [38], and two forms of the normalized difference water index (NDWI) [39, 40] (Table 2).

**Table 3:** Classification of malaria risk levels

	Trend in incidence		
	Increasing	Stable	Decreasing
Mean incidence			
Above outbreak detection threshold	High	Medium	Medium
In between	Medium	Low	Low
Below expected incidence	Medium	Low	Low

Levels were assigned based on observed malaria incidence within the early detection window, and based on predicted future malaria incidence within the early warning window

## Results

### Subsystems

#### Public health interface

The public health interface, implemented on the EPIDEMIA project website [41], serves as the primary web interface between public health users and the EPIDEMIA system (Fig. 1). The user interface provides information about the EPIDEMIA project, allows users to log in and out, upload, query, and download data, download reports, and view activity logs. Different levels of permission can be assigned by individual user accounts to allow users to upload data, download data, download the weekly reports, and access logs of upload and download requests.

To upload data, users fill in a web form to specify an epidemiological dataset and select a local Excel® workbook file containing the data in the appropriate format. After the user submits the form, the system ensures that uploaded data files contain the correct column headers and that the data values fall within acceptable ranges. If no errors are detected, the system inserts the data into the appropriate EPIDEMIA database table. If any errors are detected, the user is presented with a detailed report and asked to upload a corrected file.

To download data, users fill in another web form by selecting the desired woredas, a range of dates, and the specific epidemiological and environmental variables that they wish to download. Results are provided as a harmonized data table in which each row represents a particular woreda during a particular year and epidemiological week, and contains both epidemiological and environmental variables summarized for that location and time period.

#### Remote sensing data acquisition and processing

Remote sensing data acquisition and initial data processing and harmonization steps (Fig. 1) are performed by EASTWeb, an open-source client-based application that automatically connects to earth observation data archives and acquires, processes, and summarizes selected remote sensing datasets [42]. EASTWeb was specifically designed to facilitate the automated retrieval of remote sensing data in near real time for disease forecasting applications. A menu driven interface is used to define projects that specify the data sources to be used and the workflow steps that will be applied. EASTWeb then automates the data processing tasks by continually searching for new data online, and triggering the appropriate workflows when data become available.

The main workflow steps were similar for all remote sensing products, including the TRMM 3B42 and 3B42RT rainfall data, MODIS LST data, and MODIS NBAR data. After downloading data, EASTWeb converts files to GeoTiff raster format, mosaics adjacent tiles into a single dataset (MODIS data only), applies quality control screening to individual pixels (MODIS data only), reprojects the data into a local Universal Transverse Mercator (UTM) projection, interpolates the data to a standard 1 km grid size, crops the rasters by the boundary of the study area, and calculates a new raster for each environmental index. Next, EASTWeb summarizes the values for each index by overlaying a GIS dataset of woreda boundaries and calculating the mean value of pixels within each woreda. At this stage, the environmental data have been spatially harmonized with the epidemiological data, and the summaries are saved as tables in a PostgreSQL database. Finally, EASTWeb performs temporal harmonization on the daily rainfall indices by calculating rainfall accumulations for each WHO epidemiological week. At this point, the rainfall indices have been temporally harmonized with the epidemiological data, whereas temporal harmonization of the 8-day MODIS data is left for the data integration subsystem.

#### Data integration

Integration of the epidemiological and environmental datasets is handled by three components on the EPIDEMIA server (Fig. 1). The *epidemiological data processing* component consists of stored procedures in the MySQL database, which join the uploaded malaria data with population data by woreda and year and then calculate the derived epidemiological variables (Table 1). The *environmental data transfer* component consists of a shell script which imports any new environmental data from the EASTWeb PostgreSQL database located on the Windows sever and moves it to the MySQL database on the Linux server. This shell script is run hourly so that these databases remain synchronized, and also runs automatically whenever new epidemiological data are uploaded.

The *data unification* component uses R scripts to temporally harmonize the MODIS-derived indices and join the epidemiological and environmental datasets into a single *unified dataset*. The data processing steps completed by the data unification component include resampling the 8- and 16-day MODIS composites to 7-day WHO epidemiological weeks and estimating any missing environmental data values via linear interpolation between adjacent weeks. Additional derived environmental variables are also computed at this stage from the woreda-level summaries. These include summaries of accumulated degree-days or accumulated moisture throughout the malaria season, deviations from the long-term expectation for a particular location and time of the season, and deviations from an assumed optimal value for malaria transmission. Finally, the epidemiological and environmental datasets are joined by woreda, year, and epidemiological week to form a table containing a single unified dataset.

#### Modelling

The modelling subsystem applies statistical time series models to the unified dataset to detect outbreaks as they occur and forecast future levels of malaria transmission (Fig. 1). The subsystem runs automatically each time the data integration subsystem completes running, i.e. weekly when the system is in operational use. All modelling is performed in R and model outputs are saved as R data files. The main emphasis in this paper is on system design and information processing, so only a brief overview of the modelling techniques is provided here. The system implements early detection of outbreaks by fitting the epidemiological data for each woreda to statistical time series models to estimate (1) the expected value of each malaria indicator at each time step given the time of year and any long-term trend and (2) a threshold level indicating an unusually high value of the malaria indicator possibly consistent with an outbreak situation. This method is analogous to other widely used algorithms for outbreak detection [43]. The subsystem also models malaria incidence in each woreda as a function of season, trend, and lagged environmental variables and forecasts malaria incidence for the next 4 weeks [for similar approaches, see 13, 15, 44, 45]. To date, total malaria incidence (including *P. falciparum*, *P. vivax*, and mixed infections) has been used as the response variable in these models. However, the system can use any malaria indicator variable for modelling and forecasting.

#### Reporting

The reporting subsystem runs automatically each time the modelling subsystem completes running, i.e. weekly when the system is in operational use (Fig. 1). Each report consists of a one-page map summary of early detection and forecasting results, a set of one-page summaries for every woreda, and several pages of maps summarizing the early detection and early warning model outputs. Reports are generated as a PDF document using knitr, an engine for dynamic report generation in R [46].

**Fig. 3:**
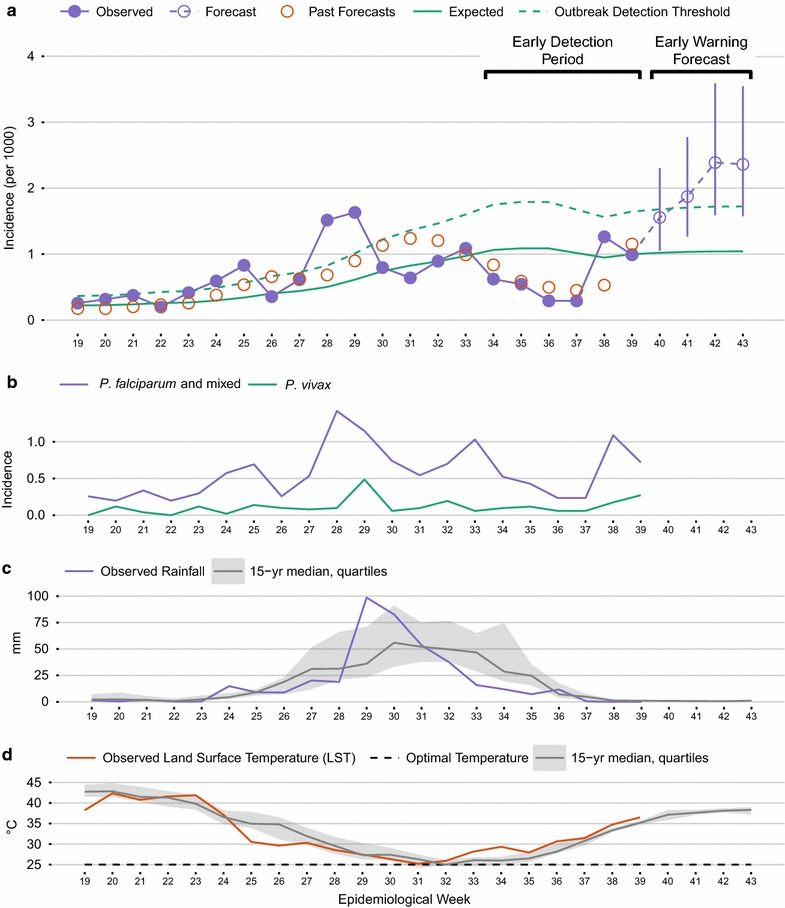
Example of the time series visualizations generated for each woreda in the weekly PDF reports. **a** Malaria control chart with observations, seasonal expected values and outbreak thresholds estimated from historical data, historical 1-week ahead forecasts, and current forecasts 4 weeks into the future; **b** observations of *Plasmodium falciparum* and *Plasmodium vivax* malaria; **c** observations of precipitation with historical climatology; **d** observations of land surface temperature with historical climatology. The data are from Abargelie woreda as of 2016 week 39

The woreda pages each contain a paragraph of automatically generated summary text and a set of four time series charts visualizing epidemiological and environmental data and model outputs over the past 21 weeks (Fig. 3). A control chart depicts observed malaria incidence, the model-derived expected incidence, the alert threshold, the 4-week forecast with 50% prediction intervals, and the historical 1-week-ahead forecasts for each of the past weeks (Fig. 3a). A second chart shows incidence of *P. falciparum* malaria and *P. vivax* malaria (Fig. 3b), while the third and fourth charts depict the observed rainfall and daytime LST along with the expected values and interquartile ranges from the reference environmental dataset (Fig. 3c, d).

**Fig. 4:**
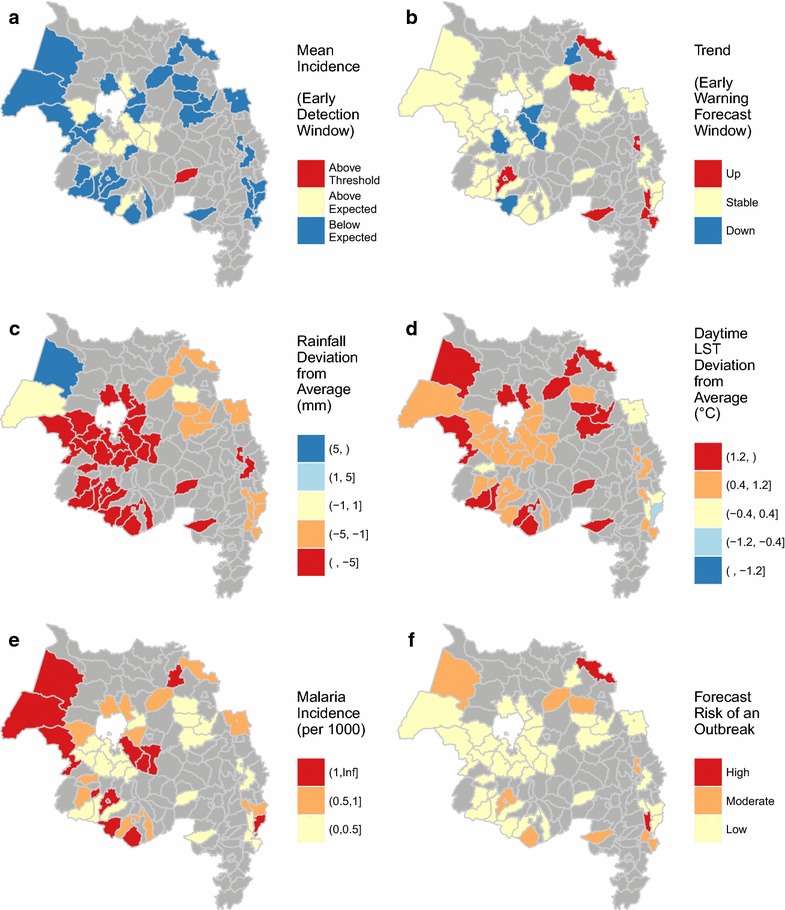
Examples of the map summaries provided in the weekly reports. **a** Malaria incidence in the early detection window; **b** malaria trend in the early warning forecast window; **c** rainfall deviation (wetter or drier than normal); **d** land surface temperature deviation (warmer or cooler than normal); **e** malaria incidence; **f** classified risk of malaria detection based on the early warning forecast (see Table 3). Data are from 2016 week 39

The woreda control charts highlight an *early detection window* (the past 6 weeks) and an *early warning forecast window* (the upcoming 4 weeks) over which data and model outputs were summarized (Fig. 3a). Within each of these summary windows, the mean observed or forecasted incidence was classified as being above the mean outbreak threshold, between the mean threshold and the mean expected incidence, or below the mean expected incidence. The overall trend in malaria incidence was also classified as increasing, decreasing, or stable for each summary window. To do this, the differences between the observed or forecasted malaria incidence and the outbreak detection threshold were calculated for each week. Linear models were then fitted to these deviations and the slope parameters were classified as positive (*β* ≥ 0.15), negative (*β* < −0.15), or in between. These classifications were useful for summarizing the weekly malaria conditions in each woreda and were mapped (Fig. 4a, b), as were the mean values of environmental indices (Fig. 4c, d) and malaria incidence (Fig. 4e) over the past 4 weeks.

Finally, composite indices of outbreak detection and outbreak forecasts were calculated for each woreda as low, medium, or high based on the classified mean incidences and the classified trends (Table 3). Maps of these indices (Fig. 4f) appeared on the first page of each report to provide a quick summary-at-a-glance of recent and upcoming outbreak risk for all woredas.

### System implementation

The EPIDEMIA system [41] was implemented on a Linux web server with 16 cores @ 3.50 GHz and 128 GB RAM. The website was written in PHP programming language for the back end; HTML, Cascading Style Sheets, JavaScript, and JQuery for the front end; and AJAX to handle asynchronous message calls. We installed Apache HTTP Server to serve the website, the database management system MySQL to store the epidemiological and environmental datasets, and the R environment for statistical computing [47] to carry out data processing and modelling tasks. These software packages are all freely available, community-driven, and open source. In addition to the primary server, the EASTWeb software was installed on a Windows server with 12 cores @ 2.50 GHz and 256 GB RAM. EASTWeb is implemented in Java programming language and uses the open source geospatial library Geospatial Data Abstraction Library (GDAL) for spatial analyses and PostgreSQL to store and manipulate the resulting data summaries.

### Operational use

The initial EPIDEMIA system co-design workshop was conducted at South Dakota State University during July 2014 and the public health interface and remote sensing data acquisition subsystem were subsequently implemented. In March 2015, a workshop was held to test the public health interface, develop a protocol for uploading the epidemiological data, and solicit feedback on a prototype weekly report. The data integration subsystem was implemented in May 2015, at which time we uploaded historical data going back to July 2012 (epidemiological data) and January 2000 (environmental data) and began regular weekly uploading of new data. In September 2015, the modelling and reporting subsystems were implemented and operational use of EPIDEMIA to produce weekly forecasts was initiated. In February and July 2016, follow-up workshops were conducted to assess system performance and solicit feedback on the next steps for improvements to the system. In addition, a steering committee of Ethiopian public health partners from ARHB, HDAMA, Bahir Dar University, and GAMBY College of Medical Sciences has met regularly to review the forecasts and provide written feedback on the accuracy of predictions, along with updates on the current malaria situation throughout the region.

As of December 2016, a total of 16,621 records have been uploaded into the EPIDEMIA system, each representing the malaria surveillance data collected from one woreda during one epidemiological week and covering the time period from July 2012-present. Malaria data were reported for 99.8% of all woreda/week combinations Also, 98.8% of reported cases in the database were confirmed by either RDT or blood film screening, a number which has increased over time from 95.1% in 2012 to over 99.9% in 2016. This situation represents a significant improvement over historical malaria surveillance in the region, where the majority of reported malaria cases were clinically diagnosed and there were considerable missing data [22]. The downloaded remote sensing data from 2000-present used 63 GB of hard drive space and the intermediary files occupied 214 GB of hard drive space. These data were distilled into a modest ~1.2 M records in tabular format, with each row containing metrics for a given environmental index, woreda, and time step. Environmental data were available for 98.3% of woreda/week combinations for LST, 99.2% for EVI, 99.3% for NDVI, SAVI, NDWI5, and NDWI6, and 100% for rainfall.

## Discussion

The EPIDEMIA system has a number of similarities to online information systems that have previously been developed for malaria and other mosquito-borne diseases, but also has several unique characteristics. Although the value of integrating climatic information and other types of remotely-sensed data into online sharing portals has been widely acknowledged [6–8], most efforts at near-real-time data sharing have focused primarily on epidemiological surveillance. For example, Eisen et al. [48] developed an online, multi-disease data management platform that handles disease case surveillance as well as entomological surveillance, but does not incorporate environmental data. Yan et al. [49] developed another online data management platform focused on syndromic surveillance in resource-constrained settings. A variety of spatial decision support systems are also being developed to rapidly collect and analyse surveillance data in support of malaria elimination efforts [e.g., 50, 51]. There have been several data management systems that support the integration of geospatial environmental datasets with mosquito-borne disease surveillance [e.g., 52–54]. However, these systems have emphasized the archiving of historical datasets rather than providing rapid access to new data. EPIDEMIA is thus distinctive in that it facilitates the rapid acquisition and processing of malaria surveillance and environmental monitoring data to produce harmonized datasets for modelling. This novel system has enabled near-real-time malaria forecasting in the Amhara region.

One of the important lessons learned through co-designing and co-implementing EPIDEMIA was that *the availability of timely, high*-*quality data is a key limiting factor* in the development of malaria early warning systems. Many previous studies have focused on the specification and testing of forecasting models, but have not explicitly considered the informatics infrastructure necessary to actually apply and test these models on a regular basis [55]. In contrast, the implementation of EPIDEMIA was based on the premise that a reliable system for providing timely, harmonized data on malaria surveillance is the critical first step in disease forecasting efforts. The review by Zinszer et al. [55] found that most evaluations of malaria forecasting models were made using historical data that, in many cases, were the same data used to fit the model parameters. In contrast, model development and evaluation is conducted as a dynamic, iterative process using the EPIDEMIA system. Because new data are acquired every week, predictions can be continuously evaluated using new, independent observations. Accuracy is also more transparent to the end users, who can carry out their own qualitative evaluations of model performance by studying patterns of model predictions in relation to the weekly observations (Fig. 3a). Thus, the development of malaria early warning systems should not be conditional upon the identification of a single “validated” model based on historical data, but should instead first focus on the development of dynamic malaria information systems as an enabling technology to support data access, model-based prediction, and continuous model evaluation and improvement. These systems must encompass not only computer software, but also networks of individuals and institutions that create a broader enabling environment to support the application of these tools.

In the EPIDEMIA project, regular face-to-face workshops facilitated communication between scientists and public health stakeholders. The steering committee with representatives of government agencies, non-governmental organizations, and universities from the study area has disseminated information about the project and gathered feedback from a wider range of stakeholders. Information obtained through these channels has been critical for co-designing and updating the system. In particular, the formal requirements analysis allowed us to identify and prioritize the system components and tailor them to end user needs. For example, the uploading interface was customized to accommodate the standard data formats used by the ARHB, allowing them to provide data at minimal burden to their staff. Concerns about internet bandwidth and accessibility resulted in an initial collaborative decision to use automatically generated PDF reports that could be disseminated as relatively small files rather than interactive, web-based data visualizations. In response to subsequent feedback, these reports were updated to incorporate a new “dashboard” format that facilitates interpretation of model forecasts in light of historical malaria trends and environmental conditions (Fig. 3), historical one-step-ahead forecasts on the control chart to facilitate model evaluation (Fig. 3a), and mapped summaries of the model predictions to provide a rapid evaluation of outbreak potential across the region (Fig. 4). Ongoing co-development efforts include testing an interactive, web-based version of the reports and evaluating the reasons for variability in model performance across woredas. These continual engagements, combined with the technical capacity to quickly modify the system in response to new feedback and suggestions, have been essential for surmounting the barriers imposed by time zone, language, and culture to achieve the successful implementation of EPIDEMIA.

Another important lesson is that projects relying on continuous retrieval of earth observation data should expect changes to the data products and have a plan for dealing with those changes. Data providers may update the algorithms for creating specific products, change the file specifications or access protocols for existing products, or cease support for some products altogether. In addition, data availability varies over time as sensors are decommissioned and replaced with new sensors that have different characteristics. Several of these issues have affected EPIDEMIA in just the first 2 years since initial design. In June 2015, the TRMM satellite was decommissioned and NASA began transitioning from the TMPA products to the newer, higher-resolution Integrated Multi-satellite Retrievals for GPM (IMERG) products. In October 2016, the TRMM archive of binary data files was decommissioned, necessitating a switch to NetCDF files. All NASA data providers including the LP DAAC and GES DISC began requiring users to register with the Earthdata Login system in August 2016 and shifted from an FTP to HTML data access protocol. To be able to respond more quickly and efficiently to these changes, the original EASTWeb software was reprogrammed as a framework with a separate plugin for each data source [56]. This design choice facilitated the modification of existing plugins and development of plugins for new data sources without having to make extensive modifications to the larger software. Upcoming changes will involve updating all MODIS plugins to handle newer versions of the data products (Collection 6) and developing a strategy for transitioning to data products from the operational VIIRS satellites once the MODIS satellites reach the end of their lifespan in the next several years.

There is potential for implementing the EPIDEMIA system in other geographic areas and applying the system to other diseases, particularly where there are strong relationships between disease transmission and environmental conditions that can serve as a basis for early warning forecasts. However, because the system also incorporates outbreak detection based on recent observations of disease cases, it still has utility in situations where the environment is not a strong predictor of epidemics such as when outbreaks are triggered by importation of a pathogen or changes in the susceptibility of the human population. The most obvious candidates for use with the EPIDEMIA system include other vector borne diseases, such as dengue, Zika, chikungunya, and West Nile, whose transmission rates are known to vary with environmental conditions affecting vector populations [57, 58]. Other types of data, particularly entomological surveillance data, could also be incorporated into the system and harmonized with the epidemiological and environmental data. For example, mosquito infection rate has been shown to be a strong predictive indicator of West Nile virus cases in the USA [57]. Entomological indicators could be used as independent predictors of malaria indices in our current modelling framework or incorporated into alternative frameworks such as dynamical models [58] or structural equation models [59]. Where available, data on malaria interventions can also be incorporated both to improve model predictions and to assess their effectiveness. There is a wide array of methods available within R to support predictive times series models [60], and this flexibility was one of the primary reasons we chose to implement the modelling subsystem entirely in that computing environment.

## Conclusions

The EPIDEMIA system has facilitated the integration of malaria surveillance data and environmental monitoring data to enable near-real-time malaria forecasts in the Amhara region of Ethiopia. As a result, it has been possible to disseminate malaria forecasts to public health partners for an extended period and engage end users in a continuous process of feedback and improvement. The development and implementation of EPIDEMIA have highlighted several considerations for anyone wishing to build such a system. Critical points include the need to develop software tools and an enabling environment to provide timely harmonized epidemiological and environmental data, the importance of continual stakeholder input throughout design, implementation, and operation of the system, and the need to be adaptable to changes in the input data. Ongoing challenges include evaluating and improving the forecasting models as new data are ingested into the system, developing more sophisticated reporting functionality such as interactive web-based visualizations, better incorporating the early detection and early warning results into public health and emergency management decision making, and ultimately transferring the tools and knowledge required to operate the system to the public health sector in Ethiopia. Once these longer-term goals have been achieved, then the system should be robust to changes in the broader social and environmental contexts of malaria and extensible to other diseases and locations.

## Abbreviations

ARHB: Amhara National Regional State Health Bureau
EVI: enhanced vegetation index
FTP: file transfer protocol
GES DISC: Goddard Earth Sciences Data and Information Services Center
GPM: Global Precipitation Mission
GUI: graphical user interface
HDAMA: Health Development and Anti-Malaria Association
IMERG: integrated multi-satellitE retrievals for GPM
IRS: indoor residual spraying
LLIN: long-lasting insecticide-treated net
LST: land surface temperature
LP DAAC: Land Processes Distributed Active Archive Center
MODIS: moderate-resolution imaging spectroradiometer
NASA: National Aeronautics and Space Administration
NDVI: normalized difference vegetation index
NDWI: normalized difference water index
PHCU: primary health care unit
RDT: rapid diagnostic test
SAVI: soil-adjusted vegetation index
TRMM: Tropical Rainfall Measurement Mission
